# “Male Depression”: Between conflict and insecurity - On the particular influence of social skills training in gender-specific treatment settings

**DOI:** 10.1192/j.eurpsy.2026.11406

**Published:** 2026-07-31

**Authors:** C. A. Penkov, K. Friedrich, J. Krieger, V. Rößner-Ruff, M. Hodel, M. Ziegenbein

**Affiliations:** Wahrendorff Klinikum, Sehnde, Germany

## Abstract

**Introduction:**

The role of interpersonal relationship behavior in the genesis and maintenance of mental disorders is well documented in the literature. Practical interactions from the field of group training for social skills (GSK) and nonviolent communication (NVC) offer effective approaches for improving social interaction skills. In the context of affective disorders, men in particular often exhibit social isolation latency, increased irritability, and interpersonal conflict potential in addition to the core symptoms of depression, which often results in unstable self-esteem. Based on the research findings, this pilot study highlights a reevaluation of the effectiveness of practical interventions with regard to improving social skills using a newly created intervention module consisting of elements of GSK and NVC, as well as the prospective effectiveness in the context of gender-specific treatment of mental illness.

**Objectives:**

Effectiveness of a newly designed therapy model consisting of GSK and NVC in gender-specific treatment of depressive disorders and implications for improved treatment approaches especially with regard to gender-specific interaction patterns and self-esteem instability.

**Methods:**

Along a newly designed therapy module consisting of eight units, patients in the intervention group at the day clinic for men at a psychiatric-psychosomatic specialist hospital complete a multimodal therapy unit lasting several weeks in a behavior therapy-oriented, group-specific treatment setting to improve their social skills. The individual modules comprise both theoretical and practical teaching approaches from the fields of GSK and NVC, using role-play situations and emotion-focused interventions for the interactive exploration of socially adaptive cognitive and behavioral patterns. Social competence factors are assessed in a pre-post design using the Social Competence Inventory (ISK-K) and correlations with self-esteem are compared with the control group. In the future, gender-specific differences in the effectiveness of multimodal social competence training between men and women will also be investigated.

**Results:**

The descriptive and inferential statistical results are presented.

**Conclusions:**

The aim of application-focused social skills training is to explore the potential influence of a new multimodal therapy unit on the modification of interactional problems and self-esteem in patients with male-specific psychopathogenic symptoms, and to derive possible implications for the treatment of mental illness in a gender-specific context.

**Disclosure of Interest:**

None Declared

